# Cardiovascular Disease and Psychiatric Comorbidity: The Potential Role of Perseverative Cognition

**DOI:** 10.1155/2009/791017

**Published:** 2009-07-22

**Authors:** Britta A. Larsen, Nicholas J. S. Christenfeld

**Affiliations:** Department of Psychology, University of California, San Diego, 9500 Gilman Drive, La Jolla, CA 92093-0109, USA

## Abstract

The high comorbidity between psychiatric disorders and cardiovascular disease has received increasing attention, yet little is known about the processes linking the two. One plausible contributing mechanism is the tendency of those with psychiatric disorders to ruminate on stressful events. This phenomenon, sometimes called perseverative cognition, can extend the psychological and physiological effects of stress, which could contribute to cardiovascular disease etiology. In this paper, we discuss the potential role of perseverative cognition in mediating the relationship between psychiatric illness and cardiovascular disease. Rumination can delay physiological recovery from acute stress, which in turn has been found to predict future cardiovascular health. This delayed recovery could act as a mechanism in the longitudinal link between worry and cardiovascular health. The cognitive inflexibility that characterizes mood and anxiety disorders may then contribute to disease not by producing greater reactivity, but instead through extending activation, increasing the risks for cardiovascular damage.

## 1. Introduction

The high comorbidity between psychiatric disorders and cardiovascular disease (CVD) has received growing attention in recent scientific literature [1–6]. Mood and anxiety disorders in particular have been linked to heart disease, with research showing that those displaying symptoms of anxiety or depression are at higher risk for cardiovascular-related morbidity and mortality [2, 7]. Those with the highest levels of anxiety have as much as a three-fold increase in risk for fatal coronary heart disease [7], and those with clinical depression have been shown to be at double the risk for cardiac incidents even as much as ten years following the onset of depression [8]. While it is clear that there is a link between these disorders, the connection itself is not well understood.

One explanation for this comorbidity is that chronic disease, such as CVD, leads to depression and anxiety, through restriction of activities, fear of impending mortality, and other consequences of debilitating disease. However, depressive symptoms have also been found to predict coronary heart disease (CHD) in cohorts not initially presenting cardiac symptoms (see [9]), suggesting a possible bidirectional model of comorbidity. Another possibility is a behavioral link, as behaviors associated with development of CVD, such as smoking, a sedentary lifestyle, and social isolation, are also associated with mood and anxiety disorders [4]. That there is a behavioral link between the two is well demonstrated, and in many cases can explain their cooccurrence. However, studies have found that these connections persist even when controlling for these important health behaviors [7]. It is possible, then, that there are further underlying traits or behaviors common to mood and anxiety disorders that could act as pathophysiological mechanisms in the development of CVD.

Another significant risk factor for CVD is psychological stress (see [3]). Stress has been linked to higher rates of morbidity and all-cause mortality [10] and is recognized as a risk factor for numerous health conditions, including cardiovascular diseases [11]. During acute stress, a number of cardiovascular changes occur, including increased heart rate and blood pressure. According to the reactivity hypothesis [12] the repeated activation of the stress response over time can result in weakened arteries, plaque buildup, and other risk factors for CVD. Consequently, those experiencing stress more frequently or to a greater degree would be at greater risk for hypertension, myocardial infarction, and other cardiovascular disorders.

While the majority of stress literature has focused on the magnitude of the stress response, it is likely that the duration of reactivity is also important. McEwen [13] has suggested that the allostatic load of a stressor, or the “total area under the curve” in which cardiovascular levels are elevated, including not just the initial response but the elevation that lingers after stress has passed, is more influential in the development of disease than the magnitude of the acute cardiovascular response. Consequently, it is important to examine not only the degree to which cardiovascular parameters are elevated above baseline, but the amount of time it takes to return to baseline levels [14, 15].

The inability to return quickly to baseline following elevated cardiovascular reactivity could therefore be an additional risk factor for cardiovascular disease. From this perspective, the “autonomic inflexibility” found in those with generalized anxiety disorder (GAD) [16] could help to explain their elevated risk for CVD. Those with GAD have been found to exhibit lower heart rate variability (HRV), suggesting lower vagal tone and a diminished influence of the parasympathetic nervous system [16]. This inflexibility could prevent recovery, increasing allostatic load and leading to future disease.

Another way of thinking about inflexibility is not in physiological terms, but as psychological flexibility. In addition to being able to return to physiological baseline, it is also important following stress to return to affective or cognitive baseline—that is, to no longer experience the cognitive or affective effects of stress. Many of the stress measures in the field, such as life event scales, measure the duration of objective stressful events in order to assess the duration of an individual's subjective affective and physiological experience of stress. However, this assumes that the experience of stress strictly coincides with the occurrence of a stressor, which is not necessarily the case. Just as autonomic inflexibility can prevent recovery from arousal, psychological inflexibility can extend brief objective stressors into long subjective ones. Consequently, the duration of a stressor is perhaps less influential for health than the duration of one's cognitive and affective response to it.

Such an approach suggests a mechanism through which the stress response can extend beyond the temporal boundaries of a stressful event, namely, prolonged cognitive activation of the stressor through rumination, worry, and anticipation. Brosschot et al. [17] have labeled this extended cognitive activation “perseverative cognition,” and have suggested that the resulting extended duration of physiological reactivity could contribute to—and may actually be necessary for—the development of serious health consequences, including cardiovascular disease, from psychological stress.

This explanation is particularly attractive when exploring cardiovascular disease's comorbidity with psychiatric disorders, since conditions such as GAD, depression, and Obsessive Compulsive Disorder (OCD) are marked by an inability to shift attention away from troubling thoughts. This is manifest both in rumination, which can extend a stressor after it has ended, and anxiety, which can induce stress before a stressor has even begun. Certainly the ability to shift focus in a timely manner and adapt to circumstances is beneficial, and it could be that the failure to do so not only marks psychiatric disorder, but also contributes to physiological disease. The cognitive intractability that is the hallmark of these psychiatric disorders could also be responsible for extending the duration of cardiovascular reactivity surrounding stressors (or, in the absence of concrete stressful events, abstract fears) and thus be deleterious for cardiovascular health.

In the pages that follow, we discuss studies that examine the concept of perseverative cognition, both as a feature of mood disorders and as a precursor to cardiovascular disease. (Some studies use the term “perseverative cognition” while others use “rumination”; in this paper the two will be used interchangeably.) We examine studies that have found connections between rumination and long-term health consequences, and also laboratory studies that test the role of perseverative cognition in delayed recovery following acute stressors, in order to explore perseverative cognition as a possible mechanism in the link between psychiatric disorders and cardiovascular health.

## 2. Perseverative Cognition and Psychopathology

As described above, those with anxiety disorders have been found to exhibit a type of autonomic inflexibility. These disorders, as well as many other psychopathologies such as depression, are also marked by a type of cognitive inflexibility; specifically, those with anxiety and depression are prone to negative fixations and an inability to shift focus away from them.

Research has shown that perseverative cognition is not only a symptom of psychopathology, but can also perpetuate it. Nolen-Hoeksema, found that trait rumination, measured using an interview to assess ruminative tendencies during negative emotion, predicted future episodes of depression and anxiety, including new onsets of depression [18]. Also, while women are at greater overall risk for depression, studies show that the risk is greater only for women high in trait rumination [19]. Consistent with this, laboratory studies show that rumination can affect mood in depressed participants. Compared to nondepressed controls, depressed participants in one study became significantly more depressed when assigned to ruminate on a stressful incident, whereas those assigned to a distraction condition showed significant increases in positive mood [20]. Furthermore, rumination has been found to extend depressive episodes [21], and learning to not ruminate appears to prevent depression [22].

Another recent study found that cognitive inflexibility may be directly caused by neurotransmitter imbalances associated with psychopathology, particularly depression, and anxiety. Compared to monkeys who underwent a sham surgery (similar incisions were made and repaired, but no actual procedure was performed in the brain), monkeys subjected to serotonin depletion in the prefrontal cortex showed an inability to relearn a reward task once it was reversed, and showed continued fixation on the previous paradigm [23]. Serotonin depletion did not, however, impede their ability to learn a new task, suggesting that this depletion did not affect cognitive abilities in general, but rather depleted their ability to shift focus and change their paradigm—behaviors typical of perseverative cognition and of the psychological disorders characterized by serotonin dysregulation.

## 3. Rumination and Long-Term Health

Several prospective studies have examined the connection between perseverative cognition and cardiovascular health, generally finding evidence of a positive link between them. One key study by Kubzansky et al. [24] measured worry across five domains (social conditions, health, finances, self-definition, and aging) in 1758 healthy men with no heart conditions and followed them for 20 years. They found that the highest level of social worry was associated with an approximately 50% increased risk for total coronary heart disease (both fatal and nonfatal), and that the risk remained highly significant when accounting for possible confounding variables. There was also evidence that worry over financial and health matters leads to elevated risk for total CHD.

It is possible that worry could be a result of rather than a cause of cardiovascular problems, though some causal role of worry in the relationship is supported by the fact that this link was stronger between worry over social conditions than worry about health conditions. If worry were only the result of, rather than a precursor to, health problems such as cardiovascular disease, one would expect the strongest relationship to be between cardiovascular disease and worry over health conditions. This, however, was not the case. This particularly points to the potential role of perseverative cognition in cardiovascular health, as social conditions are often particularly ambiguous and likely to lead to extended worry or rumination. While financial and health problems are often concrete and have definitive endpoints (such as paying off a debt or reducing one's blood pressure), social problems rarely have such concrete indicators that the trouble has passed. It may be unclear, for example, whether an argument with a friend is truly resolved, making those involved particularly prone to rumination about the situation. However, while these data show that worry can predict future health outcomes, they do not speak to whether it is the physiological effect of worrying that was the critical aspect in producing these health problems.

## 4. Rumination and Recovery from Psychological Stress

The effects of worry and rumination on recovery from stress have been investigated in a handful of laboratory studies. Generally, these have found that worry and rumination delay cardiovascular recovery from stress. Such findings at least suggest a possible explanation for the relationship between worry and CHD. Research on cardiovascular reactivity has measured responses from both the sympathetic and parasympathetic nervous systems. Roughly speaking, sympathetic response, typically measured with heart rate and blood pressure, is responsible for increased physiological arousal, while parasympathetic response, often called vagal tone, is responsible for returning the body to baseline levels following sympathetic reactivity.

As mentioned previously, several studies have examined stress reactivity and recovery in samples with clinical anxiety disorders [16]. The majority of these have assessed parasympathetic response, typically by measuring heart rate variability (HRV), which has been found to predict cardiovascular morbidity and mortality [25, 26]. The majority of these studies have found that those with clinical anxiety symptoms had reduced vagal tone and reduced heart rate variability following stressors [16, 27, 28], suggesting a delayed recovery to baseline. While these studies suggest that those with anxiety are prone to slower cardiovascular recovery, they do not address whether this is in fact due to any cognitive processes. It could be, for example, that lowered vagal tone extends the physiological effects of stress, which then causes an individual to ruminate.

Most laboratory studies with nonclinical populations have analyzed sympathetic response to stress. Several of these have found that those high in trait rumination and worry, but not necessarily meeting criteria for GAD or other psychiatric disorders, exhibit slower heart rate and blood pressure recovery following psychological stressors [29–31]. Results from this line of research are mixed, however [32, 33]. The variable results could partly be due to methods of measurement. These studies use many different measures to assess trait worry and rumination, yet none of them explicitly investigate whether participants engage in rumination following stressors. While measures of one's tendency to ruminate are informative, it seems that assessments of whether participants actually ruminate following stress in the lab could be much more useful. Such measures could both indicate the role of cognitive processes in recovery and speak to the validity of trait rumination measures. For example, common items on such measures include Likert-type scales for statements such as “I keep thinking about events that angered me for a long time” [34], which could easily be validated by assessing whether participants rating these statements as very accurate about themselves do in fact continue to think about laboratory stressors that angered them.

Another problem facing these laboratory studies is determining whether differences between groups are in fact due to a distinct recovery process, or are due to differences in initial reactivity. While emotional stressors may lead to impeded recovery, this could be a result of these stressors eliciting the greatest initial reactivity. In this case, recovery would simply be a function of reactivity rather than a distinct process influenced by separate factors. It is possible that those with anxiety and depression experience longer recovery partly because they experience greater reactivity during actual stressors.

One key study explored several of the issues stated above by orthogonally manipulating reactivity and emotionality of stressful tasks. Glynn et al. [35] monitored cardiovascular reactivity during and following four laboratory stressors. Two stressors (a cold pressor task and a shock-threat task) were meant to elicit moderate physiological reactivity, while the other two (mental arithmetic with harassment and physical exercise) were meant to elicit high physiological reactivity. Additionally, the mental arithmetic and shock threat were meant to be more emotional tasks, while the cold pressor and exercise tasks were meant to be predominantly physical stressors. Should recovery time be largely a function of reactivity magnitude, one would expect the tasks eliciting higher reactivity to also have slower recovery. Alternatively, if recovery is an affective and cognitive process rather than a purely physical process, one would expect the tasks higher in emotionality to result in slower recovery. The authors found that while the exercise and mental arithmetic with harassment tasks elicited the greatest initial arousal, it was in fact the two more emotional tasks—arithmetic and shock threat—that showed delayed recovery, supporting the view that the emotional content of the task influenced recovery more than did the initial magnitude of response.

In order to investigate further whether this continued activation was due to rumination, the authors conducted a second study in which all participants completed a mental arithmetic task with harassment. Following the stressor, half were told to relax and sit quietly, while the other half were given a distracting nonemotion task to prevent them from thinking about the stressor. Again, those who were allowed to ruminate on the task experienced continued elevation of SBP, while those who were distracted recovered quickly to baseline. Other studies have since replicated these findings, showing that cognitive distraction can expedite physiological return to baseline while allowing a subject to ruminate prevents recovery [29, 31].

These findings underscore several important points. First, they emphasize that reactivity and recovery are two distinct processes, and given that they can respond independently, the former should not be used as a proxy or a predictor for the latter. While stressors like the shock threat could be considered minimally damaging because they elicit a small initial change in CVR, they could be disproportionately deleterious for cardiovascular health because their effects are long lasting. Secondly, these findings also highlight the role of emotion in recovery from stress. Stress is typically measured in terms of the duration of the actual stressor, but if there are emotions tied to that stressor then the actual experience of stress—and resulting physiological reactivity—can continue well after the event has ended. It could be that worrying over social conditions predicted greater risk of CHD in the Kubzansky et al. study [24] because these problems were more emotionally upsetting and generated more frequent rumination than other problems like inadequate finances. Finally, these findings emphasize that the delayed recovery observed with more emotional tasks is a cognitive rather than physiological effect. While emotional events may produce different patterns of physiological arousal to nonemotional tasks, recovery from such tasks, such as the math task, is at least partially determined by cognitive focus.

Other studies have shown that the physiological effects of stress can begin well *before* the onset of an actual stressful event. While there is a good deal of research on reactivity related to general anxiety, there is relatively little research examining the effects of anticipating concrete events. Though the number of studies is small, several have shown increased cardiac activation in those anticipating stressors compared to controls (see [17]). One study also showed that physiological effects of stress can even be manifest during sleep, the night prior to a stressful event, suggesting both that perseverative cognition needs not to be conscious, and that its health consequences may extend to impaired sleep quality [36]. The data connecting anticipation of stressors and adverse health outcomes are, however, extremely limited.

## 5. Cardiovascular Recovery and Long-Term Health Outcomes

While the allostatic load theory predicts that extended stress activation, such as that experienced during rumination, leads to long-term negative health, longitudinal studies with health outcomes are needed to verify the approach. Several prospective studies have examined whether recovery from acute laboratory stressors predicts morbidity and mortality. The results from several of these studies show quite strikingly that recovery, even when measured over only several minutes, can predict health years later. Two of these studies found that participants who took longer to recover from acute cardiovascular reactivity were more likely to develop hypertension in the years following [37, 38]. Stewart and France found that this effect held even when controlling for initial reactivity, emphasizing again that recovery and reactivity are distinct risk factors. Treiber et al. [39] also found that both reactivity and recovery following a stressor independently predicted heart rate and blood pressure four years later after controlling for initial values. Another laboratory study investigated the role of reactivity and recovery in healthy college students whose parents either did or did not have heart disease [40]. The authors found no difference in initial reactivity between the groups, but found that those who had two parents with heart disease displayed slower blood pressure recovery. This suggests that delayed recovery may act as a mechanism in the heritability of heart disease. That is, children may not inherit “heart disease” from their parents, but in some cases inherit cardiovascular tendencies such as delayed recovery that eventually lead to cardiovascular disease.

Two other studies also found that slow recovery from acute reactivity (in this case due to exercise) predicted all-cause mortality five and six years later (see [41, 42], resp.). Of course, these studies do not speak to the influence of perseverative cognition; cardiovascular reactivity was due to physical exercise rather than psychological stress, and there are myriad health-related factors, such as physical fitness, that could affect both recovery and mortality risk independently. Importantly, however, the Cole et al. study found that recovery predicted mortality even when controlling for initial health status, including weight, blood pressure, and other factors that could affect cardiovascular health. This suggests that delayed recovery, whether due to physical or psychological causes, could have health consequences independent of other cardiovascular risk factors. 

While these studies suggest a connection between recovery and health, clearly there are gaps that must be filled with additional research. Specifically, prospective studies are needed to evaluate whether delayed cardiovascular recovery from psychological stressors, particularly due to rumination, can predict future mortality. It is unclear at this point whether rumination can lead to heart disease in otherwise physically healthy subjects, or whether some physical predisposition is also needed for disease to develop. Such studies could suggest a model whereby perseverative cognition contributes to physical disease, which would likely be in one of two ways. First, it is possible that those with physiological flexibility—that is, normal sympathetic and parasympathetic responses—engage in perseverative cognition, and by doing so extend sympathetic responses, decrease vagal tone, and consequently damage the cardiovascular system. In this case, perseverative cognition would be a key causal factor. Alternatively, it is possible that certain people experience autonomic inflexibility due purely to physiological rather than psychological factors. The resulting extended activation could both trigger cognitive fixation on the stressor and cause damage to the cardiovascular system. Perseverative cognition in this model could play some role in extending the response, but it would serve more as a marker than a cause of future disease. Deciphering between these models would further elucidate which individual differences act as risks for heart disease.

**Figure 1:**
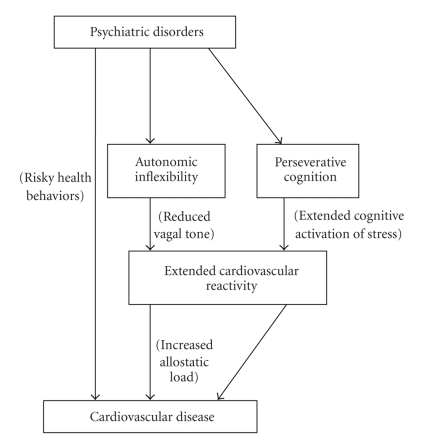
This informal theoretical model shows the potential role of perseverative cognition in linking psychiatric disorders and cardiovascular disease. While behavioral and physiological mechanisms have been highlighted (the left and middle pathways, resp.), this figure shows an additional cognitive pathway on the right, in which cardiovascular reactivity is extended due to cognitive fixation on stressful experiences.

Figure 1  shows a simplified view of the comorbidity relationship. Clearly many of the links are bidirectional and more interconnected than shown, but the figure is designed for illustrative purposes. The left path illustrates the well-established behavioral link mentioned previously. The central path illustrates the more physiologically based connection, in which psychopathology is associated with deficiencies in homeostatic mechanisms, leading to extended elevated cardiovascular reactivity following stress. The focus of this paper has been to highlight the pathway on the right, which illustrates a cognitive pathway linking these phenomena. Extended activation in this case is not due to a physiological deficit, but rather to cognitive fixation on negative experiences and worries. These pathways are by no means mutually exclusive; rather, it is likely that all three (plus additional mechanisms) play a role in the relationship between psychopathology and cardiovascular disease. 

## 6. The Role of Flexibility in Health

The studies discussed above emphasize an often overlooked individual difference affecting health, namely, flexibility. As the ability to adapt is an essential aspect of survival, the importance of this phenomenon should not be surprising. With much attention devoted to the duration of actual stressors, it has been chronic stressors that have been deemed most deleterious for health. These presumably result in the longest lasting physiological, cognitive, and affective negative consequences. However, the effects of a chronic stressor should be much less dire for individuals able to regulate negative emotions, adjust appraisals of threat, and alter ineffective coping strategies. Along the same lines, those with inflexible coping responses could experience negative affective, cognitive, and physiological consequences from even brief stressors. In fact, it has been suggested that coping flexibility, or the ability to shift between coping strategies, predicts effective coping responses to stress better than any particular coping strategy [43]. Folkman and Lazarus [44] emphasized that coping is a shifting process and found that within a healthy population coping strategies fluctuated markedly not only between but also within individuals.

While the research on flexibility's impact on health is limited, there is some evidence that it predicts better overall mental and physical health (see [45]). One laboratory study found that emotional flexibility and regulation in college freshmen predicted lower levels of distress 1.5 years later [46]. Flexibility could also be a mechanism through which social support confers health benefits [47]. It is possible that supportive others could offer new coping strategies or different appraisals, or act as distractions to prevent rumination.

Flexibility may also be a common theme in various coping strategies that have been associated with better health. The Constructive Anger Behavior-Verbal scale (CAB-V), for example, measures the degree to which people express anger verbally in order to understand “the other person's point of view” and “strive to achieve a new way of perceiving and dealing with the anger situation” [48]. As opposed to those who expressed anger simply to alert someone that they were angry, those high in constructive anger expression had lower resting blood pressure, even when controlling for hypertension risk factors and psychosocial measures. Writing about traumatic experiences in order to organize and understand them has also been found to improve mental and physical health [49, 50], which could be partially due to the writing process offering new interpretations and shifting appraisals of events. Finally, cognitive behavioral therapy (CBT), which focuses on cognitive restructuring and reappraisals of events, has been shown to be an effective treatment for depression and anxiety. Furthermore, several large-scale studies, such as the Enhancing Recovery in Coronary Heart Disease Patients (ENRICHD) study, have used this method of cognitive restructuring specifically to address depression and anxiety in patients with cardiovascular disease [51]. These studies have found that CBT can effectively address psychopathology in these patients, and that in some cases it also leads to improved cardiac health [52].

While these studies do not examine flexibility specifically, they do show that coping strategies that focus on enhancing understanding and embracing new perspectives, which could both be construed as features of cognitive flexibility, are associated with better health. 

## 7. Conclusions

As shown in the studies above, inflexibility, whether cognitive, emotional, or physiological, can be damaging for mental and physical health. It is possible that the high comorbidity between cardiovascular disease and psychiatric disorders is attributable to a general state of inflexibility, leading to rumination, worry, obsessions, low heart rate variability and vagal tone, and extended sympathetic arousal. Additionally, it could be that inflexibility in any one of these areas perpetuates it in another. Perseverative cognition, for example, has been shown to cause both extended physiological arousal [35], and extended and/or more severe depression [21, 22].

While the studies discussed here suggest a role of perseverative cognition in linking psychiatric disorders and cardiovascular disease, clearly there are questions remaining to be answered. Rumination has been shown to delay recovery from stress, a condition which predicts future cardiovascular health, yet it remains to be established that long-term health outcomes can in fact be predicted by differences in rumination. Similarly, while several studies found evidence of an autonomic inflexibility in those with GAD, it is uncertain whether a cognitive inflexibility, or inability to shift cognitions to more constructive paths, might also play a role.

As perseverative cognition has been found to extend physiological activity in a way believed to be deleterious for health, a better understanding of the situational and individual factors that contribute to this phenomenon is clearly important. Generally, cognitive fixation suggests a lack of adaptability that threatens one's ability to thrive in a physical and social world.
